# The Durability of Antibody Responses of Two Doses of High-Dose Relative to Two Doses of Standard-Dose Inactivated Influenza Vaccine in Pediatric Hematopoietic Cell Transplant Recipients: A Multi-Center Randomized Controlled Trial

**DOI:** 10.1093/cid/ciad534

**Published:** 2023-10-06

**Authors:** Jennifer E Schuster, Lubna Hamdan, Daniel E Dulek, Carrie L Kitko, Einas Batarseh, Zaid Haddadin, Laura S Stewart, Anna Stahl, Molly Potter, Herdi Rahman, Spyros A Kalams, Claire E Bocchini, Elizabeth A Moulton, Susan E Coffin, Monica I Ardura, Rachel L Wattier, Gabriela Maron, Michael Grimley, Grant Paulsen, Christopher J Harrison, Jason L Freedman, Paul A Carpenter, Janet A Englund, Flor M Munoz, Lara Danziger-Isakov, Andrew J Spieker, Natasha B Halasa, for the Pediatric HCT Flu Study, Rakesh Goyal, Joanne Thurber, Rendie McHenry, Margaret Bender, Shari Barto, Michael Russo, Lauren Shoemaker, Kenny Truong, Christopher Dvorak, Kim J Allison, Swati Naik, Christopher Williams, Samantha Blum, Kirsten Lacombe, Hannah Smith

**Affiliations:** Department of Pediatrics, Children's Mercy Kansas City, Kansas City, Missouri, USA; Department of Pediatrics, Vanderbilt University Medical Center, Nashville, Tennessee, USA; Department of Pediatrics, Vanderbilt University Medical Center, Nashville, Tennessee, USA; Department of Pediatrics, Vanderbilt University Medical Center, Nashville, Tennessee, USA; Department of Pediatrics, Vanderbilt University Medical Center, Nashville, Tennessee, USA; Department of Pediatrics, Vanderbilt University Medical Center, Nashville, Tennessee, USA; Department of Pediatrics, Vanderbilt University Medical Center, Nashville, Tennessee, USA; Department of Pediatrics, Vanderbilt University Medical Center, Nashville, Tennessee, USA; Department of Pediatrics, Vanderbilt University Medical Center, Nashville, Tennessee, USA; Department of Pediatrics, Vanderbilt University Medical Center, Nashville, Tennessee, USA; Department of Medicine, Vanderbilt University Medical Center, Nashville, Tennessee, USA; Department of Pediatrics, Division of Infectious Diseases, Baylor College of Medicine, and Texas Children's Hospital, Houston, Texas, USA; Department of Pediatrics, Division of Infectious Diseases, Baylor College of Medicine, and Texas Children's Hospital, Houston, Texas, USA; Department of Pediatrics, Perelman School of Medicine, University of Pennsylvania, Philadelphia, Pennsylvania, USA; Department of Pediatrics, Division of Infectious Diseases & Host Defense, Nationwide Children's Hospital and The Ohio State University, Columbus, Ohio, USA; Department of Pediatrics, University of California San Francisco and Benioff Children's Hospital – San Francisco, San Francisco, California, USA; Department of Infectious Diseases, Children's, St. Jude Children's Research Hospital, Memphis, Tennessee, USA; Department of Pediatrics, University of Cincinnati College of Medicine, Cincinnati Children's Hospital Medical Center, Cincinnati, Ohio, USA; Department of Pediatrics, University of Cincinnati College of Medicine, Cincinnati Children's Hospital Medical Center, Cincinnati, Ohio, USA; Department of Infectious Diseases, University of Missouri at Kansas City, Kansas City, Missouri, USA; Department of Pediatrics, Perelman School of Medicine, University of Pennsylvania, Philadelphia, Pennsylvania, USA; Department of Pediatrics, University of Washington and Seattle Children's Research Institute, Seattle, Washington, USA; Department of Pediatrics, University of Washington and Seattle Children's Research Institute, Seattle, Washington, USA; Department of Pediatrics, Division of Infectious Diseases, Baylor College of Medicine, and Texas Children's Hospital, Houston, Texas, USA; Department of Molecular Virology and Microbiology, Baylor College of Medicine, Houston, Texas, USA; Department of Pediatrics, University of Cincinnati College of Medicine, Cincinnati Children's Hospital Medical Center, Cincinnati, Ohio, USA; Department of Biostatistics, Vanderbilt University Medical Center, Nashville, Tennessee, USA; Department of Pediatrics, Vanderbilt University Medical Center, Nashville, Tennessee, USA

**Keywords:** pediatrics, stem cell recipients, influenza, vaccination, high dose

## Abstract

**Background:**

Our previous study established a 2-dose regimen of high-dose trivalent influenza vaccine (HD-TIV) to be immunogenically superior compared to a 2-dose regimen of standard-dose quadrivalent influenza vaccine (SD-QIV) in pediatric allogeneic hematopoietic cell transplant (HCT) recipients. However, the durability of immunogenicity and the role of time post-HCT at immunization as an effect modifier are unknown.

**Methods:**

This phase II, multi-center, double-blinded, randomized controlled trial compared HD-TIV to SD-QIV in children 3–17 years old who were 3–35 months post-allogeneic HCT, with each formulation administered twice, 28–42 days apart. Hemagglutination inhibition (HAI) titers were measured at baseline, 28–42 days following each dose, and 138–222 days after the second dose. Using linear mixed effects models, we estimated adjusted geometric mean HAI titer ratios (aGMR: HD-TIV/SD-QIV) to influenza antigens. Early and late periods were defined as 3–5 and 6–35 months post-HCT, respectively.

**Results:**

During 3 influenza seasons (2016–2019), 170 participants were randomized to receive HD-TIV (n = 85) or SD-QIV (n = 85). HAI titers maintained significant elevations above baseline for both vaccine formulations, although the relative immunogenic benefit of HD-TIV to SD-QIV waned during the study. A 2-dose series of HD-TIV administered late post-HCT was associated with higher GMTs compared to the early post-HCT period (late group: A/H1N1 aGMR = 2.16, 95% confidence interval [CI] = [1.14–4.08]; A/H3N2 aGMR = 3.20, 95% CI = [1.60–6.39]; B/Victoria aGMR = 1.91, 95% CI = [1.01–3.60]; early group: A/H1N1 aGMR = 1.03, 95% CI = [0.59–1.80]; A/H3N2 aGMR = 1.23, 95% CI = [0.68–2.25]; B/Victoria aGMR = 1.06, 95% CI = [0.56–2.03]).

**Conclusions:**

Two doses of HD-TIV were more immunogenic than SD-QIV, especially when administered ≥6 months post-HCT. Both groups maintained higher titers compared to baseline throughout the season.

**Clinical Trials Registration:**

NCT02860039.

## Teaser

In pediatric hematopoietic cell transplant (HCT) recipients, 2 doses of high-dose trivalent influenza vaccine were more immunogenic than 2 doses of standard-dose quadrivalent influenza vaccine, especially when administered ≥6 months post-HCT. Influenza-specific titers remained higher than baseline for all antigens.

## BACKGROUND

Influenza infection can cause severe illness, particularly in immunocompromised individuals [1–3]. In a 3-year retrospective observational cohort study of 1560 pediatric hematopoietic cell transplantation (HCT) recipients, influenza accounted for 11% of respiratory viral infection-associated hospitalizations, with 21% of influenza-infected HCT recipients having lower respiratory tract involvement at diagnosis, 31% requiring subsequent respiratory support, and 10% dying [4]. The mainstay for the prevention of influenza disease in pediatric HCT recipients is standard-dose inactivated influenza vaccination. However, pediatric HCT recipients have reduced humoral immune responses to vaccination when compared to age-matched healthy children, suggesting that alternative influenza vaccine regimens are needed [5].

Given the paucity of influenza vaccine studies in pediatric HCT recipients, current influenza vaccine guidelines mainly rely on extrapolation from adult populations, and there is no consensus on the ideal timing post-HCT, formulation, and number of doses [6–11]. For example, the 2013 Infectious Diseases Society of America vaccination guidelines recommend a 2-dose regimen of standard-dose (SD) inactivated influenza vaccine for children 6 months to <9 years if it is their first time receiving influenza vaccine [6]; other experts recommend considering a second dose—regardless of age—especially when the first vaccine is given <6 months post-HCT [8]. In contrast, the 2013 European Guidelines for the Prevention and Management of Influenza in HCT Patients recommend inactivated influenza vaccine be given as early as 3 months post-HCT, and a second dose of vaccine 3–4 weeks later is advised, noting that the second dose may only have marginal benefit [7].

Phase I studies demonstrated high-dose (HD) influenza vaccine to be safe and immunogenic in some high-risk non-HCT pediatric populations [9–11] and more immunogenic than SD in high-risk adult populations [12]. Previously, we reported that a 2-dose series of high-dose trivalent inactivated influenza vaccine (HD-TIV) induces a greater immune response against both influenza A antigens in pediatric allogeneic HCT patients compared to a 2-dose series of standard-dose quadrivalent inactivated influenza vaccine (SD-QIV) [13]. The goal of this study was to further evaluate the influence of host factors and timing of initial influenza immunization post-HCT on immunogenicity, characterize the durability of antibodies, and determine the advantage of HD-TIV throughout the influenza season.

## METHODS

### Trial Design and Participants

This phase II, multi-center, double-blinded, randomized controlled trial of pediatric HCT recipients was conducted over 3 influenza seasons (2016–17, 2017–18, and 2018–19) at 9 US study sites: Vanderbilt University Medical Center (TN) as the leading site; University of California San Francisco Benioff Children's Hospital—San Francisco (CA), Children's Mercy Kansas City (MO), Cincinnati Children's Hospital (OH), Nationwide Children's Hospital (OH), Children's Hospital of Philadelphia (PA), St. Jude Children's Research Hospital (TN), Texas Children's Hospital (TX), and Seattle Children's Hospital (WA). (*Pediatric HCT Flu Study; ClinicalTrials.gov number, NCT02860039*).

Eligible participants were 3–17 years old and 3–35 months post-allogeneic HCT. Participants with graft versus host disease (GVHD) were eligible if their disease and GVHD therapy were stable for at least 4 weeks prior to enrollment. Further details regarding inclusion and exclusion criteria and the schedule of events are previously published [13].

Participants were randomized on a 1:1 basis to receive either 2 doses of HD-TIV or 2 doses of SD-QIV with a target interval of 28–42 days between vaccine doses (at the time of this study, the high-dose formulation of the quadrivalent vaccine was not available). Randomization was blocked and stratified by site and time post-HCT. Moreover, randomization of participants <12 months post-HCT was stratified by GVHD status, systemic steroid use, receipt of alemtuzumab or anti-thymocyte globulin pre-transplant, cord blood or haploidentical transplant, or post-HCT cyclophosphamide. For participants ≥12 months post-HCT, randomization was stratified by GVHD and/or systemic steroid use. We determined that a sample size of n = 160 evaluable participants would be required to achieve 80% power for the trial's primary goal of comparing 2 doses of HD-TIV to 2 doses of SD-QIV (this calculation was based on an analysis of a between-group difference of 20% in proportions achieving a titer ≥1:40) [13].

The study was reviewed and approved by the Institutional Review Board at each of the study sites. All parents/guardians provided written informed consent; participants provided assent per site-specific IRB requirements by age. Study data were collected and randomization was performed using allocation concealment in REDCap electronic data capture tools hosted at Vanderbilt University Medical Center [14, 15].

### Vaccine

Vaccines were provided by Sanofi (Swiftwater, PA) and investigational pharmacies at each site dispensed study vaccines per randomization code in a blinded manner. SD-QIV contained 15 µg hemagglutinin from each strain (A/H1N1, A/H3N2, B/Victoria, B/Yamagata). HD-TIV contained 60 µg of the hemagglutinin from each strain except for B/Yamagata.

### Study Procedures

Vaccines were administered as 0.5 mL intramuscular deltoid injections given at a target interval of 28–42 days apart (visits 1 and 2). Per protocol, complete blood count, CD4^+^/CD8^+^/CD19^+^ cells, total immunoglobulin M (IgM) and immunoglobulin G (IgG) concentrations, and serological and cellular assays were scheduled for collection prior to administration of each vaccine, as well as 28–42 days (visit 3) and 138–222 days (visit 4) following the second vaccine. During site-specific influenza seasons, nasal swabs were obtained at each study visit regardless of symptoms, and/or if participants had influenza-like illness.

### Immunogenicity Assays

Serum samples were frozen at each site, shipped to Vanderbilt and then bulk-shipped to Sanofi Global Clinical Immunology for blinded hemagglutination inhibition (HAI) testing for each vaccine-specific antigen [9]. When blood volume was insufficient, HAI testing of influenza A antigens was prioritized.

### Influenza Surveillance

Active influenza surveillance occurred during each site's local influenza season, defined as ≥10% of clinical or research laboratory samples testing positive for influenza for 2 consecutive weeks by either molecular or rapid testing [16, 17]. When a participant had influenza-like illness, communication occurred, and nasal swabs were collected and shipped to Vanderbilt University Medical Center for testing by Luminex NxTAG RPP® plus influenza B lineage typing by singleplex polymerase chain reaction [18].

### Statistical Analyses

In all model-based analyses, missing data (including missing values resulting from incorrect vaccine doses) were addressed using multiple imputation by chained equations (M = 300 iterations). A total of 4 participants died during the post-second dose follow-up period; data collected from these participants were included in analyses, although missing values for variables due to death were not imputed.

#### Descriptive Analyses

Within each group, we generated baseline (ie, visit 1) descriptive statistics as median (interquartile range [IQR]) for continuous variables and absolute and relative frequencies for categorical variables. All descriptive analyses were based on participants receiving at least 1 vaccine dose.

HAI titers to each antigen were summarized within each vaccine group at each visit as: geometric mean titer (GMT), proportion achieving titers ≥1:40 (a proxy for seroprotection), proportion achieving ≥4-fold rise from visit 1 (a proxy for seroconversion), and geometric mean fold-rise from baseline (GMFR: eg, HD-TIV at visit 2, 3, or 4/HD-TIV at visit 1) [13].

#### Predictors of Immunogenicity and Durability

We compared immunogenicity between vaccine groups using linear mixed models with log-transformed HAI titer as the outcome; we included log-transformed baseline titer, time post-HCT, CD4^+^ count, CD19^+^ count, absolute lymphocyte count (ALC), GVHD, and malignancy as adjustment covariates, as well as participant- and site-specific random effects. From each model, we obtained estimates and 95% confidence intervals (CIs) for adjusted geometric mean ratios (aGMR: eg, HD-TIV at visit 2/SD-QIV at visit 2) at each follow-up time. Note that results on B/Yamagata are reported as a control because this strain was included in SD-QIV but not in HD-TIV. To identify baseline covariates predictive of post-dose 2 titers, we fit an analogous model for visits 3 and 4 outcomes. We did not perform this analysis for B/Yamagata due to concerns regarding lack of sufficient power (participants in the HD-TIV group did not receive this antigen and could not be included in this analysis).

#### Time Post-HCT as an Effect Modifier

As an exploratory subgroup analysis, we stratified estimates of aGMRs by time post-HCT at baseline (early: 3–5 months, and late: 6–35 months). Additionally, we investigated time post-transplant as a continuous modifier of vaccine immunogenicity, including a continuous spline-interaction within vaccine group. The natural cubic spline featured 3 knots (chosen at 4, 12, and 24 months).

## RESULTS

### Study Participants

**Figure 1.**
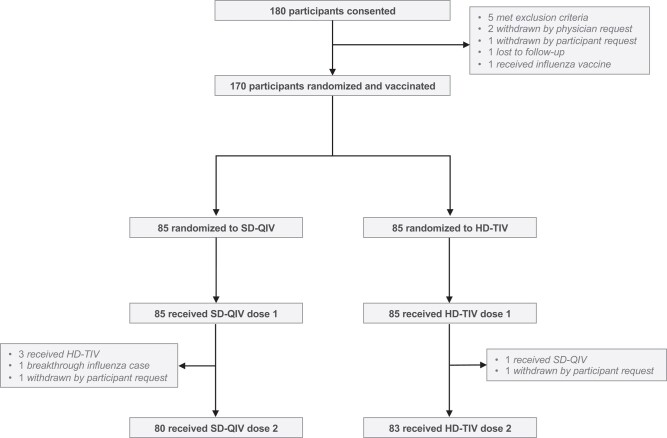
Enrollment, randomization, and vaccine status. A total of 180 participants were consented, among whom 170 were subsequently randomized and vaccinated. Among the 85 participants randomized to receive SD-QIV, 80 (94%) received both doses; among the 85 participants randomized to receive HD-TIV, 83 (98%) received both doses. Abbreviations: HD-TIV, high-dose trivalent influenza vaccine; SD-QIV, standard-dose quadrivalent influenza vaccine.

A total of 181 children were enrolled; 170 were randomized, received at least 1 vaccine, and were considered evaluable for analysis (n = 85 received SD-QIV and n = 85 received HD-TIV, Figure 1); characteristics of participants were previously described [13]. Sixty-eight (40.0%) participants were vaccinated at 3–5 months post-HCT, 50 (29.4%) at 6–11 months, and 52 (30.6%) at 12–35 months. Demographic, transplant-related, and clinical characteristics for the overall cohort and each vaccine group were largely similar, although the median time post-transplant was higher in the SD-QIV group (Table 1). Distribution by sites is reported in Supplementary Table 1.

**Table 1.** Cohort Demographics and Clinical Characteristics, Stratified by Treatment arm

	All (N = 170)	Control (SD-QIV) (N = 85)	Experimental (HD-TIV) (N = 85)
*Demographics—no. (%)*			
Age at enrollment, years			
Mean (SD)	10.8 (4.3)	10.6 (4.4)	11.0 (4.2)
Median (IQR)	10.9 (7.0, 14.3)	10.6 (7.0, 14.2)	11.8 (7.1, 14.3)
Minimum, maximum	3.1, 18.0	3.1, 18.0	3.3, 18.0
Sex, male	94 (55.3)	49 (57.7)	45 (52.9)
Race			
White	117 (68.8)	62 (72.9)	55 (64.7)
Black/African American	31 (18.2)	12 (14.1)	19 (22.4)
Asian	6 (3.5)	2 (2.4)	4 (4.7)
American Indian/Alaskan Native	1 (0.6)	1 (1.2)	0 (0)
Other/unknown	15 (8.8)	8 (9.4)	7 (8.2)
Hispanic	36 (21.2)	17 (20.0)	19 (22.4)
*Transplant Characteristics—no. (%)*			
Indication for transplant			
Malignant	96 (56.5)	45 (52.9)	51 (60.0)
AML/ANLL	35/96 (36.5)	21/45 (46.7)	14/51 (27.5)
ALL	40/96 (41.7)	16/45 (35.6)	24/51 (47.1)
CML	4/96 (4.2)	2/45 (4.4)	2/51 (3.9)
MDS/MPN	8/96 (8.3)	2/45 (4.4)	6/51 (11.8)
Other	9/96 (9.4)	4/45 (8.9)	5/51 (9.8)
Non-malignant	74 (43.5)	40 (47.1)	34 (40.0)
Severe aplastic anemia	25/74 (33.8)	14/40 (35.0)	11/34 (32.4)
Inherited erythrocyte abnormalities	31/74 (41.9)	15/40 (37.5)	16/34 (47.1)
Immune system disorders	6/74 (8.1)	2/40 (5.0)	4/34 (11.8)
Fanconi anemia	6/74 (8.1)	4/40 (10.0)	2/34 (5.9)
Other^a^	6/74 (8.1)	5/40 (12.5)	1/34 (2.9)
Time from transplant to enrollment, months			
Median (IQR)	7.8 (4.3, 13.4)	9.2 (5.0, 15.9)	6.0 (4.1, 12.2)
≥3 to <5 m	68 (40.0)	25 (29.4)	43 (50.6)
≥6 to <12 m	50 (29.4)	30 (35.3)	20 (23.5)
≥12 to <36 m	52 (30.6)	30 (35.3)	22 (25.9)
Related donor	79 (46.5)	40 (47.1)	39 (45.9)
Stem cell source			
Bone marrow	108 (63.5)	55 (64.7)	53 (62.4)
Peripheral blood	46 (27.1)	22 (25.9)	24 (28.2)
Umbilical cord blood	13 (7.7)	7 (8.2)	6 (7.1)
Multiple sources	3 (1.8)	1 (1.2)	2 (2.4)
Condition preparation regimen			
Myeloablative	123/168 (73.2)	64/84 (76.2)	59/84 (70.2)
Reduced-intensity or non-myeloablative	42/168 (25.0)	19/84 (22.6)	23/84 (27.4)
Other	3/168 (1.8)	1/84 (1.2)	2/84 (2.4)
Total body irradiation	68/168 (40.5)	29/84 (34.5)	39/84 (46.4)
T-cell depletion	75/169 (44.4)	39 (45.9)	36/84 (42.9)
GVHD status at vaccine 1			
Acute	8 (4.7)	3 (3.5)	5 (5.9)
Chronic	15 (8.8)	8 (9.4)	7 (8.2)
GVHD history			
Acute	45 (26.5)	24 (28.2)	21 (24.7)
Chronic	23 (13.5)	13 (15.3)	10 (11.8)
Both	9 (5.3)	6 (7.1)	3 (3.5)
Rituximab post-transplant^b^	12/136 (8.8)	6/67 (9.0)	6/69 (8.7)
Recipient CMV status, negative	60 (35.3)	29 (34.1)	31 (36.5)
*Baseline lab values at visit 1—median (IQR)*			
WBC (10^3^/μL)	5.5 (4.4, 7.8)	5.6 (4.3, 8.4)	5.5 (4.4, 7.2)
ANC (10^3^/μL)	2.8 (2.1, 4.1)	2.8 (2.0, 4.5)	2.7 (2.1, 4.0)
ALC (10^3^/μL)	1.7 (1.0, 2.6)	1.9 (1.1, 2.9)	1.4 (1.0, 2.4)
CD4^+^ count (cells/μL)	385 (205, 752)	491 (230, 810)	310 (190, 569)
CD8^+^ count (cells/μL)^c^	434 (197, 738)	442 (211, 734)	426 (192, 741)
CD19^+^ count (cells/μL)	424 (211, 762)	443 (249, 882)	423 (185, 644)
Hemoglobin (g/dL)	12.3 (11.3, 13.5)	12.6 (11.5, 13.5)	12.1 (11.3, 13.3)
Platelets (10^3^/μL)	206 (149, 264)	209 (160, 267)	204 (143, 261)
Quantitative IgG (mg/dL)	718 (573, 965)	705 (583, 972)	735 (568, 943)
Quantitative IgM^d^ (mg/dL)	54 (34, 93)	51 (32, 93)	56 (35, 92)

Abbreviations: ALC, absolute leukocyte count; ALL, acute lymphoblastic leukemia; AML, acute myelogenous leukemia; ANLL, acute non-lymphocytic leukemia; ANC, absolute neutrophil count; CML, chronic myelogenous leukemia; GVHD, graft versus host disease; HD-TIV, high-dose trivalent influenza vaccine; IgG, immunoglobulin G; IQR, interquartile range; MDS, myelodysplastic syndrome; MPN, myeloproliferative neoplasms; N, number of participants enrolled who received at least 1 vaccination; SD, standard deviation; SD-QIV, standard-dose quadrivalent influenza vaccine; WBC, white blood count.

^a^GATA2 haploinsufficiency (n = 1 for SD-QIV), Hurlers syndrome (n = 1 for SD-QIV), Inherited abnormalities of platelets (n = 1 for HD-TIV), Inherited disorders of metabolism (n = 1 for SD-QIV), Sanfilippo Syndrome-Type A (n = 1 for SD-QIV), X-linked lymphoproliferative syndrome (n = 1 for SD-QIV).

^b^Rituximab prescription was not recorded for first year participants, and the information was unknown for 34/170 total subjects.

^c^CD8 result missing for 1 HD-TIV subject.

^d^Quantitative immunoglobulin M (IgM) was missing for 2 SD-QIV subjects.

### Antibody Responses and Durability by Vaccine Group

**Figure 2.**
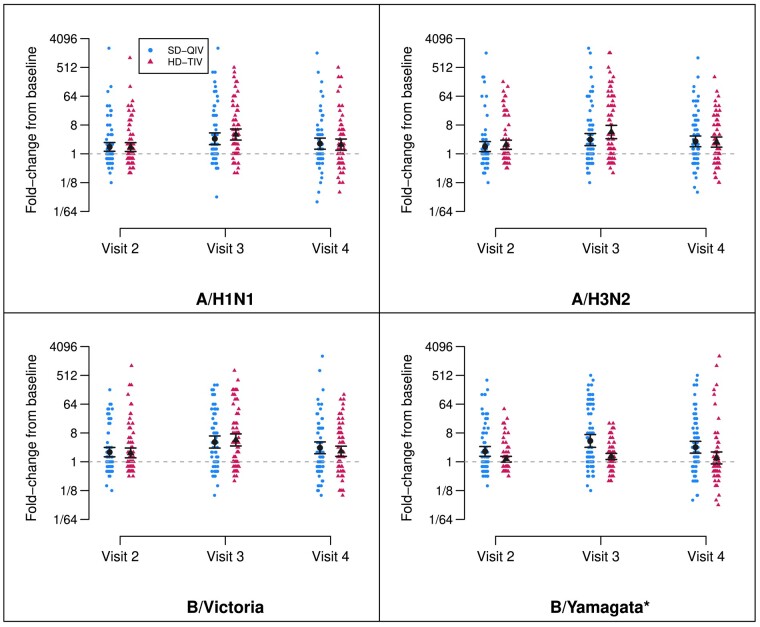
Fold-rises by vaccine group and dose. Depiction of titer fold-rises from baseline (visit 1, prior to the first dose), shown by randomization group (SD-QIV and HD-TIV) for each antigen and each follow-up visit. The estimated GMFR and 95% confidence intervals are depicted in black. Visit 2 titers are measured at a target window of 28–42 d following the first dose (prior to the second dose), visit 3 titers are measured at a target window of 28–42 d following the second dose, and visit 4 titers are measured at a target window of 138–222 d following the second dose. All GMFRs were significantly different from zero across time points for both vaccine groups with the exception of B/Yamagata for HD-TIV at visit 4 (*B/Yamagata was not included in HD-TIV). Abbreviations: GFMR, geometric mean fold-rise; HD-TIV, high-dose trivalent influenza vaccine; SD-QIV, standard-dose quadrivalent influenza vaccine.

For both vaccine groups, the visit 2 and 3 GMTs were significantly higher as compared to baseline for each A/H1N1, A/H3N2, and B/Victoria antigens (Table 2; Figure 2). At visit 4 (138–222 days after vaccine dose 2), GMTs remained higher than baseline for all 3 antigens. Other measures of immunogenicity further indicated higher titers from baseline after each vaccine dose (Supplementary Table 2 and Table 2).

**Table 2.** Point Estimates and 95% CIs for Group-specific Geometric Mean Fold-rises (GMFRs) and Adjusted Geometric Mean Ratios (aGMRs, Comparing High-dose [HD-TIV] to Standard-dose [SD-QIV]), Shown for Each Antigen at Each Follow-up Visit

	GMFR (95% CI)		aGMR (95% CI)
	SD-QIV (n = 85)	HD-TIV (n = 85)	(HD-TIV/SD-QIV)
A/H1N1			
Visit 2	1.65 (1.20–2.27)	1.63 (1.18–2.25)	1.19 (0.78–1.85)
Visit 3	2.97 (1.92–4.58)	4.02 (2.63–6.13)	1.65 (1.06–2.57)
Visit 4	2.08 (1.37–3.17)	1.94 (1.24–3.03)	1.21 (0.77–1.90)
A/H3N2			
Visit 2	1.68 (1.17–2.42)	1.93 (1.38–2.69)	1.33 (0.84–2.11)
Visit 3	2.79 (1.78–4.37)	4.82 (2.90–8.01)	2.11 (1.32–3.38)
Visit 4	2.46 (1.65–3.68)	2.33 (1.56–3.49)	1.44 (0.88–2.33)
B/Victoria			
Visit 2	2.01 (1.42–2.83)	1.89 (1.32–2.71)	1.11 (0.71–1.73)
Visit 3	4.16 (2.65–6.53)	4.84 (3.06–7.67)	1.46 (0.93–2.31)
Visit 4	2.74 (1.75–4.28)	2.13 (1.42–3.20)	1.09 (0.68–1.74)
B/Yamagata ^a^			
Visit 2	2.11 (1.48–3.02)	1.21 (0.98–1.48)	0.66 (0.43–1.02)
Visit 3	4.50 (2.80–7.21)	1.47 (1.16–1.85)	0.40 (0.26–0.63)
Visit 4	2.85 (1.83–4.46)	1.32 (0.82–2.12)	0.70 (0.44–1.13)

Visit 2 titers are measured at a target window of 28–42 days following the first dose (prior to the second dose). Visit 3 titers are measured at a target window of 28–42 days following the second dose. Visit 4 titers are measured at a target window of 138–222 days following the second dose.

Abbreviations: CI, confidence interval; HD-TIV, high-dose trivalent influenza vaccine; SD-QIV, standard-dose quadrivalent influenza vaccine.

^a^B/Yamagata is not included in HD-TIV.

The aGMRs for A/H1N1, A/H3N2, and B/Victoria antigens comparing HD-TIV to SD-QIV at all four visits are reported in Table 2. Compared to SD-QIV, the HD-TIV group had significantly higher estimated GMTs at visit 3 for A/H1N1 and A/H3N2 as previously reported [13]. Although the HD-TIV group trended toward higher GMTs than SD-QIV at both visit 2 and 4, we did not identify significant differences in the 3 antigens.

### Predictors of Post-dose 2 Antibody Titers

Covariate-specific aGMRs for predictors of visit 3 and visit 4 HAI titers to each antigen included participant, vaccine, and immune-related factors (Table 3). In addition to higher baseline HAI titers, we found higher CD19^+^ count to be predictive of higher post-dose 2 titers for A/H1N1, A/H3N2, and B/Victoria, at both visits 3 and 4. Higher CD4^+^ count and lower ALC were significantly associated with higher titers for A/H3N2 at visit 4. History of GVHD was significantly associated with lower titers to A/H3N2 at visit 3. Of note, longer time from HCT to first immunization was more strongly associated with higher titers at visit 3, although we did not see such evidence at visit 4.

**Table 3.** Point Estimates and 95% CIs for aGMRs Associated With Each Model Covariate for Visit 3 (28–42 d Post-dose 2) and 4 (Approximately 6 Months Post-dose 2) HAI Titers to Each Influenza Antigen

Visit 3	A/H1N1	A/H3N2	B/Victoria
Vaccine group (HD-TIV)	1.60 (1.02–2.50)	1.86 (1.10–3.13)	1.44 (0.90–2.31)
log_2_-baseline titer	1.24 (1.12–1.38)	1.32 (1.20–1.46)	1.24 (1.11–1.38)
Age (y)	0.97 (0.91–1.03)	1.01 (0.94–1.07)	0.97 (0.91–1.03)
Time post-HCT (m)	1.06 (1.02–1.10)	1.06 (1.01–1.11)	1.06 (1.02–1.10)
CD4^+^ count	0.98 (0.87–1.11)	1.12 (0.98–1.28)	1.02 (0.90–1.15)
CD19^+^ count	1.09 (1.02–1.17)	1.12 (1.04–1.21)	1.12 (1.05–1.21)
ALC (100/μL)	0.97 (0.93–1.01)	0.95 (0.90–1.00)	0.97 (0.93–1.02)
GVHD history (Yes)	0.82 (0.50–1.34)	0.53 (0.30–0.92)	0.92 (0.56–1.53)
Malignant (Yes)	0.86 (0.51–1.43)	0.93 (0.52–1.64)	0.86 (0.52–1.44)

Visit 4	A/H1N1	A/H3N2	B/Victoria
Vaccine group (HD-TIV)	1.22 (0.76–1.99)	1.47 (0.93–2.34)	1.07 (0.67–1.71)
log_2_-baseline titer	1.30 (1.16–1.46)	1.47 (1.34–1.60)	1.29 (1.16–1.44)
Age (y)	0.99 (0.93–1.06)	1.05 (0.99–1.12)	1.01 (0.95–1.08)
Time post-HCT (m)	1.03 (0.99–1.07)	1.02 (0.98–1.06)	1.02 (0.98–1.07)
CD4^+^ count	1.05 (0.92–1.19)	1.25 (1.11–1.40)	1.11 (0.98–1.25)
CD19^+^ count	1.10 (1.02–1.18)	1.09 (1.01–1.17)	1.13 (1.05–1.22)
ALC (100/μL)	0.96 (0.91–1.00)	0.95 (0.91–0.99)	0.96 (0.92–1.00)
GVHD history (Yes)	0.86 (0.50–1.46)	0.97 (0.58–1.63)	0.84 (0.50–1.40)
Malignant (yes)	0.58 (0.33–1.00)	0.78 (0.46–1.32)	0.97 (0.58–1.64)

Abbreviations: aGMR, adjusted geometric mean ratio; ALC, absolute leukocyte count; CI, confidence interval; GVHD, graft versus host disease; HAI, hemagglutination inhibition; HCT, hematopoietic cell transplant; HD-TIV, high-dose trivalent influenza vaccine.

### Antibody Responses by Post-transplant Period

**Figure 3.**
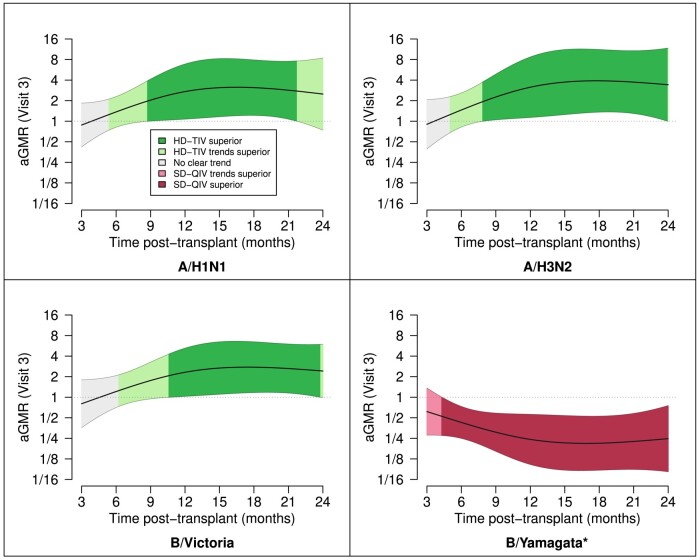
Continuous time post-HCT as a moderator of vaccine-induced immunogenicity. Depicted are the aGMRs and corresponding point-wise 95% confidence intervals as a function of time post-HCT (months). We only depict this over the first 24 m due to sparsity of data outside that time range. The strength of evidence is shaded based on superiority (*P* < 0.05), trending superiority (*P* > 0.05 but aGMR > 1.25 or aGMR < 0.8), or no clear trend (all other cases); *B/Yamagata was not included in HD-TIV. Abbreviations: aGMR, adjusted geometric mean ratio; HD-TIV, high-dose trivalent influenza vaccine; SD-QIV, standard-dose quadrivalent influenza vaccine.

Group- and visit-specific GMFRs and corresponding 95% CIs in the early (3–5 months) and late (6–35 months) post-HCT periods, along with stratum-specific aGMRs, are depicted in Table 4. Additional immunogenicity endpoints by early and late post-HCT periods are summarized in Supplementary Table 3. In summary, participants vaccinated late post-HCT had markedly higher post-vaccine rises in HAI titers compared to participants vaccinated early post-HCT, regardless of dose. Notably, we found that in the late post-HCT period, HD-TIV was associated with significantly higher GMTs not only for A/H1N1 and A/H3N2 but also for B/Victoria. In our analysis of continuous time post-transplant as a possible effect modifier, we found the immunogenic benefit of HD-TIV relative to SD-QIV starts to become evident after approximately 6 months post-HCT, with maximum benefit after approximately 1 year (Figure 3).

**Table 4.** Point Estimates and 95% CIs for aGMRs Comparing High-dose (HD-TIV) to Standard-dose (SD-QIV), Shown for Each Antigen at Each Follow-up Visit and Stratified by Time post-HCT

3–5 m post-HCT			
	GMFR (95% CI)		aGMR (95% CI)
	SD-QIV (n = 25)	HD-TIV (n = 43)	HD-TIV/SD-QIV
A/H1N1			
Visit 2	0.84 (0.70–1.00)	0.83 (0.70–0.99)	0.98 (0.57–1.70)
Visit 3	2.03 (1.07–3.85)	1.95 (1.31–2.89)	1.03 (0.59–1.80)
Visit 4	1.35 (0.69–2.63)	0.97 (0.60–1.58)	0.92 (0.51–1.67)
A/H3N2			
Visit 2	0.75 (0.63–0.89)	1.02 (0.79–1.32)	1.26 (0.70–2.29)
Visit 3	1.52 (0.80–2.86)	1.84 (1.13–3.00)	1.23 (0.68–2.25)
Visit 4	0.85 (0.54–1.33)	0.99 (0.65–1.52)	1.34 (0.71–2.54)
B/Victoria			
Visit 2	0.81 (0.64–1.03)	0.99 (0.72–1.36)	1.01 (0.53–1.92)
Visit 3	1.74 (0.89–3.40)	2.12 (1.29–3.49)	1.06 (0.56–2.03)
Visit 4	1.02 (0.54–1.91)	1.03 (0.62–1.71)	0.98 (0.49–1.97)
B/Yamagata ^a^			
Visit 2	0.99 (0.72–1.35)	0.84 (0.72–0.98)	0.79 (0.43–1.43)
Visit 3	2.50 (1.14–5.45)	1.09 (0.82–1.45)	0.44 (0.24–0.81)
Visit 4	1.22 (0.61–2.42)	0.82 (0.48–1.43)	0.65 (0.33–1.29)

6–35 m post-HCT			
	GMFR (95% CI)		aGMR (95% CI)
	SD-QIV (n = 61)	HD-TIV (n = 41)	HD-TIV/SD-QIV
A/H1N1			
Visit 2	2.21 (1.44–3.40)	3.24 (1.85–5.67)	1.37 (0.74–2.56)
Visit 3	3.53 (2.02–6.16)	9.37 (4.65–18.9)	2.16 (1.14–4.08)
Visit 4	2.49 (1.48–4.19)	4.04 (2.04–7.99)	1.48 (0.77–2.86)
A/H3N2			
Visit 2	2.39 (1.47–3.90)	3.71 (2.13–6.46)	1.31 (0.67–2.55)
Visit 3	3.68 (2.08–6.53)	14.9 (6.73–33.1)	3.20 (1.60–6.39)
Visit 4	3.87 (2.36–6.35)	5.77 (3.31–10.1)	1.68 (0.83–3.39)
B/Victoria			
Visit 2	2.99 (1.91–4.67)	3.67 (2.04–6.61)	1.13 (0.61–2.08)
Visit 3	6.23 (3.58–10.8)	12.7 (6.38–25.4)	1.91 (1.01–3.60)
Visit 4	4.21 (2.44–7.26)	4.61 (2.70–7.88)	1.20 (0.63–2.29)
B/Yamagata ^a^			
Visit 2	2.93 (1.83–4.69)	1.75 (1.23–2.48)	0.51 (0.27–0.94)
Visit 3	5.87 (3.29–10.5)	2.08 (1.48–2.94)	0.31 (0.16–0.60)
Visit 4	4.10 (2.39–7.04)	2.17 (1.02–4.63)	0.68 (0.35–1.33)

Visit 2 titers are measured at a target window of 28–42 days following the first dose (prior to the second dose). Visit 3 titers are measured at a target window of 28–42 days following the second dose. Visit 4 titers are measured at a target window of 138–222 days following the second dose.

Abbreviations: aGMR, adjusted geometric mean ratio; CI, confidence interval; GMFR, geometric mean fold-rise; GVHD, graft versus host disease; HCT, hematopoietic cell transplant; HD-TIV, high-dose trivalent influenza vaccine; SD-QIV, standard-dose quadrivalent influenza vaccine.

^a^B/Yamagata is not included in HD-TIV.

### Laboratory Confirmed Influenza Cases

We identified a total of 13 individuals (7.6%) with laboratory-confirmed influenza infections (Supplementary Table 4); seven cases in the HD-TIV group were detected, of which 4 were due to B/Yamagata, which was not included in HD-TIV. The other 3 cases were influenza A: 2 were A/H3N2 cases and 1 was an A/untypable case. In the SD-QIV group, 6 influenza cases were detected, including 1 A/H1N1 case, 3 A/H3N2 cases, 1 B/Victoria case, and 1 B/Yamagata case.

## DISCUSSION

Our multi-center double-blinded phase II randomized controlled trial of pediatric HCT recipients demonstrated that a 2-dose series of HD-TIV was more immunogenic for influenza A than a 2-dose series of SD-QIV, and the relative benefit is more pronounced in patients who received their first influenza immunization at ≥6 months post-HCT. Although the benefit of HD-TIV compared with SD-QIV in end-of-season immunogenicity remains unclear, HAI titers remained significantly increased from baseline at the end of influenza season for both HD-TIV and SD-QIV groups.

To our knowledge, our study is the first to evaluate a 2-dose regimen of HD-TIV in HCT recipients of any age. Previous studies have evaluated the effect of only 1 dose of HD influenza vaccine. In a phase I single center study of 44 adult HCT recipients (median time post-HCT: 7.9 months), a single dose of HD-TIV produced higher GMT (GMR = 6.9) and a higher percentage of individuals with protective titers to A/H3N2 (81% vs. 36%) compared to a single dose of SD-TIV [9]. In a prior phase I safety and immunogenicity trial in 38 pediatric solid organ transplant recipients, 1 dose of HD-TIV also produced better immune responses with a higher percentage of seroconversion to A/H3N2 (56% vs 13%) and higher GMT (GMR = 2.5) for A/H1N1 compared to 1 dose of SD-TIV [10]. In our study, the HD-TIV group met each of the three criteria for the historical World Health Organization biological standards for influenza vaccine immunogenicity after 2 doses for all three antigens: (1) >40% achieving seroconversion (four-fold rise), (2) a GMFR from baseline of >2.5, and (3) >70% achieving seroprotection (HAI titer ≥1:40); whereas the SD-QIV group did not meet the criteria for seroconversion for both influenza A antigens. Importantly, neither group met seroconversion criteria after 1 dose, highlighting the importance of a second dose to function as a booster and increase immune response. By visit 4, geometric mean antibody titers did not revert to their baseline values in either group, but there is not sufficient evidence of longevity in the relative benefit of high dose (ie, 6 months after the second dose). This relative benefit is likely most important during the time of peak influenza circulation and may not be needed for the entire influenza season, but the duration of this remains unknown and is an important direction of future study. These data suggest HD inactivated influenza vaccination is a practical strategy to overcome suboptimal immune responses and highlights the importance of a 2-dose influenza vaccine regimen in pediatric HCT recipients.

Time from transplantation to vaccination and absolute CD19^+^ count have previously been identified as strong predictors of influenza vaccine immunogenicity [19], similar to our data. In the current study, the greatest benefit of HD-TIV relative to SD-QIV was in participants receiving their first influenza immunization ≥6 months post-HCT with no significant difference in immune response in participants vaccinated with HD-TIV <6 months post-HCT. However, 2 doses of either vaccine were more immunogenic than 1 dose. Therefore, additional protection is needed for this high-risk group such as vaccinating close contacts and early use of antivirals with exposure to influenza-positive individuals.

We note that there was a high degree of variation in HAI titers at baseline, with higher baseline titers observed among participants early post-HCT. This could be due to previous history of influenza vaccination or IVIG administration with passive transfer of HAI antibody. Baseline HAI titer served as an important predictor of post-vaccination HAI titer, even with adjustment for time post-HCT. This form of heterogeneity in our sample therefore improved our power to detect associations and served as a strength. Furthermore, this highlights the importance of determining the baseline immunogenicity status of each patient and evaluating the results of the intervention.

This study has some limitations. The HD-TIV product used in this trial did not include B/Yamagata while SD-QIV did; however, prior studies noted that an additional B-antigen does not interfere with the immune responses of the other 3, thereby mitigating challenges surrounding generalizability. Biological standards of vaccine immunogenicity are a proxy of protection but not an established correlate of protection in this population. Therefore, active influenza surveillance was conducted. Moreover, this trial was not powered to determine the efficacy of HD-TIV compared to SD-QIV for the prevention of influenza infection. However, we report fewer breakthrough influenza cases in the HD-TIV group when restricted to the influenza strains included in both vaccines. Lastly, the study was not powered to detect end of season durability differences between the 2 groups.

In summary, this phase II safety and immunogenicity trial demonstrated that 2 doses of HD-TIV compared to 2 doses SD-QIV in pediatric HCT recipients 3–35 months post-HCT resulted in greater antibody responses, especially for influenza A antigens and for participants ≥6 months post-HCT. With the current preference for HD-QIV in the elderly population, clinicians could consider 2 doses of HD-QIV as an option in the pediatric HCT population based on these data. Because influenza causes substantial morbidity and mortality in this high-risk population, optimization of vaccine strategies is critical and the administration of two doses of HD inactivated influenza vaccine could be a practical strategy to overcome poor immunogenicity associated with standard, single-dose influenza vaccination.

## Supplementary Data

Supplementary materials are available at *Clinical Infectious Diseases* online. Consisting of data provided by the authors to benefit the reader, the posted materials are not copyedited and are the sole responsibility of the authors, so questions or comments should be addressed to the corresponding author. The members of the Pediatric HCT Flu Study Group are listed below:

^1^Children's Mercy Kansas City: Rakesh Goyal, Joanne Thurber

^2^Vanderbilt University Medical Center: Rendie McHenry, Margaret Bender, Shari Barto

^3^Children's Hospital of Philadelphia: Dr Michael Russo

^4^Nationwide Children's Hospital: Lauren Shoemaker

^5^University of California San Francisco Benioff Children's Hospital: Kenny Truong, Dr Christopher Dvorak

^6^St. Jude Children's Research Hospital: Kim J. Allison

^7^Baylor School of Medicine, Texas Children's Hospital: Swati Naik, Christopher Williams, and the Texas Children's Hospital Research Resources Office.

^8^Cincinnati Children's Hospital: Samantha Blum

^9^Seattle Children's Research Institute: Kirsten Lacombe, Hannah Smith

## Supplementary Material

ciad534_Supplementary_Data
