# Supplementary material for: The Durability of Antibody Responses of Two Doses of High-Dose Relative to Two Doses of Standard-Dose Inactivated Influenza Vaccine in Pediatric Hematopoietic Cell Transplant Recipients: A Multi-Center Randomized Controlled Trial
Source: Clin Infect Dis. 2023 Oct 6;78(1):217–26. doi: 10.1093/cid/ciad534 (PMC10810702; doi:10.1093/cid/ciad534)
Supplement: ciad534_Supplementary_Data [file ciad534_supplementary_data.docx]

**SUPPLEMENTARY APPENDIX**

**Table S1.** Enrollment by sites stratified by control and experimental group.

|  | All  (N=170) | Control  (SD-QIV)  (N=85) | Experimental  (HD-TIV)  (N=85) |  |
| --- | --- | --- | --- | --- |
| *Site of enrollment – no. (%)* |  |  |  |  |
| Vanderbilt University Medical Center (Nashville) | 13 (7.7) | 5 (5.9) | 8 (9.4) |  |
| Seattle Children’s Hospital | 13 (7.7) | 7 (8.2) | 6 (7.1) |  |
| Children’s Hospital of Philadelphia | 16 (9.4) | 10 (11.8) | 6 (7.1) |  |
| Children’s Mercy Hospital (Kansas City) | 18 (10.6) | 11 (12.9) | 7 (8.2) |  |
| Cincinnati Children’s Hospital | 39 (22.9) | 20 (23.5) | 19 (22.4) |  |
| Nationwide Children’s Hospital (Columbus) | 11 (6.5) | 5 (5.9) | 6 (7.1) |  |
| St. Jude Children’s Research Hospital (Memphis) | 10 (5.9) | 3 (3.5) | 7 (8.2) |  |
| Texas Children’s Hospital (Houston) | 42 (24.7) | 21 (24.7) | 21 (24.7) |  |
| UCSF Benioff Children’s Hospital (San Francisco) | 8 (4.7) | 3 (3.5) | 5 (5.9) |  |
| N: Number of participants enrolled who received at least one vaccination | | | | |

**Table S2**. Point estimates and 95% CIs for geometric mean HAI titer (GMT), titer ≥1:40, and proportion with ≥4-fold rise from baseline (not applicable for visit 1) for each vaccine regimen (SD-QIV and HD-TIV), stratified for each antigen. Also included is the ratio of the observed values (n) to the number of evaluable subjects (N) at each visit. Visit 1 titers measured at baseline (prior to first vaccine dose), visit 2 titers are measured at a target window of 28-42 days following the first dose (prior to the second dose), visit 3 titers are measured at a target window of 28-42 days following the second dose, and visit 4 titers are measured at a target window of 138-222 days following the second dose.

|  |  | A/H1N1 | | A/H3N2 | | B/Victoria | | B/Yamagata | |
| --- | --- | --- | --- | --- | --- | --- | --- | --- | --- |
| Visit |  | SD-QIV (N=85) | HD-TIV (N=85) | SD-QIV (N=85) | HD-TIV (N=85) | SD-QIV (N=85) | HD-TIV (N=85) | SD-QIV (N=85) | HD-TIV (N=85) |
| 1 | n/N (%) | 85/85 (100%) | 85/85 (100%) | 85/85 (100%) | 85/85 (100%) | 84/85 (99%) | 85/85 (100%) | 85/85 (100%) | 85/85 (100%) |
|  | GMT | 55 (38-78) | 64 (45-91) | 77 (52-115) | 62 (42-92) | 43 (30-61) | 41 (29-56) | 54 (38-77) | 59 (42-82) |
|  | % ≥1:40 | 66% (55%-75%) | 69% (59%-79%) | 64% (53%-73%) | 65% (54%-74%) | 52% (42%-63%) | 56% (46%-67%) | 65% (54%-74%) | 67% (57%-76%) |
| 2 | n/N (%) | 83/85 (98%) | 83/85 (98%) | 83/85 (98%) | 83/85 (98%) | 82/85 (96%) | 83/85 (98%) | 83/85 (98%) | 83/85 (98%) |
|  | GMT | 88 (62-126) | 104 (70-156) | 136 (89-207) | 123 (78-191) | 88 (60-128) | 79 (54-117) | 116 (82-164) | 71 (51-98) |
|  | % ≥1:40 | 73% (63%-82%) | 70% (60%-79%) | 73% (63%-23%) | 71% (61%-80%) | 67% (56%-77%) | 70% (60%-79%) | 81% (71%-88%) | 71% (61%-80%) |
|  | % ≥4-fold rise | 20% (13%-30%) | 20% (13%-30%) | 14% (8%-23%) | 22% (14%-31%) | 23% (15%-33%) | 24% (16%-34%) | 25% (17%-35%) | 12% (6%-20%) |
| 3 | n/N (%) | 80/84 (94%) | 76/85 (89%) | 80/84 (94%) | 76/85 (89%) | 79/84 (94%) | 76/85 (89%) | 80/84 (94%) | 76/85 (89%) |
|  | GMT | 167 (116-241) | 275 (196-387) | 235 (158-351) | 327 (208-515) | 188 (131-270) | 225 (154-329) | 269 (190-382) | 97 (71-133) |
|  | % ≥1:40 | 82% (73%-90%) | 92% (85%-97%) | 86% (78%-93%) | 88% (78%-93%) | 85% (76%-92%) | 91% (83%-96%) | 90% (82%-95%) | 82% (72%-89%) |
|  | % ≥4-fold rise | 31% (22%-42%) | 42% (31%-53%) | 31% (22%-42%) | 41% (30%-52%) | 46% (35%-57%) | 46% (35%-57%) | 41% (31%-52%) | 20% (12%-30%) |
| 4 | n/N (%) | 76/84 (90%) | 70/85 (82%) | 77/84 (92%) | 70/85 (82%) | 77/84 (92%) | 70/85 (82%) | 77/84 (92%) | 70/85 (82%) |
|  | GMT | 110 (76-158) | 125 (83-187) | 186 (127-271) | 152 (98-235) | 113 (79-162) | 97 (68-138) | 152 (108-213) | 86 (55-133) |
|  | % ≥1:40 | 78% (67%-86%) | 76% (65%-85%) | 86% (77%-92%) | 79% (6%-87%) | 78% (68%-86%) | 79% (68%-87%) | 82% (72%-89%) | 67% (56%-77%) |
|  | % ≥4-fold rise | 29% (20%-40%) | 31% (21%-43%) | 35% (25%-46%) | 36% (25%-47%) | 38% (28%-49%) | 34% (24%-46%) | 42% (31%-53%) | 19% (11%-29%) |

**Table S3**. Point estimates and 95% CIs for geometric mean HAI titer (GMT), titer ≥1:40, and proportion with ≥4-fold rise from baseline (not applicable for visit 1) for each vaccine regimen (SD-QIV and HD-TIV), stratified for each antigen. Also included is the ratio of the observed values (n) to the number of evaluable subjects (N) at each visit. Visit 1 titers measured at baseline (prior to first vaccine dose), visit 2 titers are measured at a target window of 28-42 days following the first dose (prior to the second dose), visit 3 titers are measured at a target window of 28-42 days following the second dose, and visit 4 titers are measured at a target window of 138-222 days following the second dose.

| **3-5 months post-HCT** | | A/H1N1 | | A/H3N2 | | B/Victoria | | B/Yamagata | |
| --- | --- | --- | --- | --- | --- | --- | --- | --- | --- |
| Visit* |  | SD-QIV (N=25) | HD-TIV (N=43) | SD-QIV (N=25) | HD-TIV (N=43) | SD-QIV (N=25) | HD-TIV (N=43) | SD-QIV (N=25) | HD-TIV (N=43) |
| 1 | n/N (%) | 25/25 (100%) | 43/43 (100%) | 25/25 (100%) | 43/43 (100%) | 25/25 (100%) | 43/43 (100%) | 25/25 (100%) | 43/43 (100%) |
|  | GMT | 149 (93-240) | 109 (67-175) | 203 (107-382) | 112 (67-188) | 99 (59-164) | 64 (42-98) | 128 (78-210) | 120 (83-173) |
|  | % ≥1:40 | 88% (72%-97%) | 81% (68%-91%) | 80% (62%-92%) | 77% (63%-88%) | 76% (57%-90%) | 72% (58%-84%) | 88% (72%-97%) | 88% (77%-96%) |
| 2 | n/N (%) | 25/25 (100%) | 42/43 (98%) | 25/25 (100%) | 42/43 (98%) | 25/25 (100%) | 42/43 (98%) | 25/25 (100%) | 42/43 (98%) |
|  | GMT | 125 (72-215) | 91 (54-156) | 151 (80-288) | 119 (68-208) | 80 (45-142) | 65 (39-107) | 126 (78-204) | 102 (69-149) |
|  | % ≥1:40 | 84% (67%-95%) | 67% (52%-80%) | 80% (62%-92%) | 76% (62%-87%) | 76% (57%-90%) | 69% (54%-82%) | 88% (72%-97%) | 81% (67%-91%) |
|  | % ≥4-fold rise | 0.0% (n/a) | 5% (0.8%-14%) | 0.0% (n/a) | 2% (0.1%-10%) | 0.0% (n/a) | 7% (2%-18%) | 4% (0.2%-16%) | 2% (0.1%-10%) |
| 3 | n/N (%) | 25/25 (100%) | 41/43 (95%) | 25/25 (100%) | 41/43 (95%) | 25/25 (100%) | 41/43 (95%) | 25/25 (100%) | 41/43 (95%) |
|  | GMT | 303 (180-508) | 240 (153-377) | 307 (171-550) | 230 (129-412) | 172 (88-335) | 150 (89-250) | 320 (173-591) | 147 (104-208) |
|  | % ≥1:40 | 96% (84%-100%) | 88% (76%-95%) | 92% (77%-99%) | 83% (70%-92%) | 84% (67%-95%) | 85% (73%-94%) | 96% (84%-100%) | 93% (82%-98%) |
|  | % ≥4-fold rise | 24% (10%-43%) | 27% (15%-42%) | 20% (8%-38%) | 20% (9%-33%) | 24% (10%-43%) | 29% (17%-44%) | 24% (10%-43%) | 12% (5%-24%) |
| 4 | n/N (%) | 22/25 (88%) | 36/43 (84%) | 23/25 (92%) | 36/43 (84%) | 23/25 (92%) | 36/43 (84%) | 23/25 (92%) | 36/43 (84%) |
|  | GMT | 193 (105-354) | 107 (59-195) | 178 (96-328) | 103 (56-190) | 93 (49-178) | 68 (40-116) | 167 (94-297) | 97 (56-167) |
|  | % ≥1:40 | 86% (68%-96%) | 72% (56%-85%) | 91% (76%-98%) | 72% (56%-85%) | 74% (54%-89%) | 72% (56%-85%) | 78% (59%-92%) | 69% (53%-83%) |
|  | % ≥4-fold rise | 23% (9%-43%) | 17% (7%-31%) | 9% (2%-25%) | 14% (5%-28%) | 17% (6%-36%) | 19% (9%-34%) | 26% (11%-46%) | 14% (5%-28%) |
| **6-35 months post-HCT** | | A/H1N1 | | A/H3N2 | | B/Victoria | | B/Yamagata | |
| Visit* |  | SD-QIV (N=60) | HD-TIV (N=42) | SD-QIV (N=60) | HD-TIV (N=42) | SD-QIV (N=60) | HD-TIV (N=42) | SD-QIV (N=60) | HD-TIV (N=42) |
| 1 | n/N (%) | 60/60 (100%) | 42/42 (100%) | 60/60 (100%) | 42/42 (100%) | 59/60 (98%) | 42/42 (100%) | 60/60 (100%) | 42/42 (100%) |
|  | GMT | 36 (23-55) | 37 (23-59) | 52 (32-83) | 34 (20-59) | 30 (20-46) | 25 (16-40) | 38 (25-58) | 28 (17-45) |
|  | % ≥1:40 | 57% (44%-69%) | 57% (42%-71%) | 57% (44%-69%) | 52% (37%-67%) | 42% (30%-55%) | 40% (27%-56%) | 55% (42%-67%) | 45% (31%-60%) |
| 2 | n/N (%) | 58/60 (97%) | 41/42 (98%) | 58/60 (97%) | 41/42 (98%) | 57/60 (95%) | 41/42 (98%) | 58/60 (97%) | 41/42 (98%) |
|  | GMT | 76 (48-119) | 119 (65-219) | 130 (76-222) | 126 (62-256) | 92 (57-148) | 97 (53-177) | 111 (70-176) | 49 (29-81) |
|  | % ≥1:40 | 69% (56%-80%) | 73% (58%-85%) | 71% (58%-81%) | 66% (51%-79%) | 63% (50%-75%) | 71% (56%-83%) | 78% (66%-87%) | 61% (46%-75%) |
|  | % ≥4-fold rise | 29% (19%-42%) | 37% (23%-52%) | 21% (12%-32%) | 41% (27%-57%) | 33% (22%-46%) | 41% (27%-57%) | 34% (23%-47%) | 22% (11%-36%) |
| 3 | n/N (%) | 55/59 (92%) | 35/42 (83%) | 55/59 (92%) | 35/42 (83%) | 54/59 (92%) | 35/42 (83%) | 55/59 (92%) | 35/42 (83%) |
|  | GMT | 128 (80-203) | 323 (193-542) | 208 (124-350) | 495 (247-991) | 197 (128-302) | 364 (216-613) | 249 (162-382) | 60 (36-99) |
|  | % ≥1:40 | 76% (64%-86%) | 97% (88%-100%) | 84% (72%-92%) | 91% (79%-98%) | 85% (74%-93%) | 97% (88%-100%) | 87% (77%-94%) | 69% (52%-82%) |
|  | % ≥4-fold rise | 35% (23%-48%) | 60% (43%-75%) | 36% (24%-49%) | 66% (49%-80%) | 56% (42%-68%) | 66% (49%-80%) | 49% (36%-62%) | 29% (15%-45%) |
| 4 | n/N (%) | 54/59 (92%) | 34/42 (81%) | 54/59 (92%) | 34/42 (81%) | 54/59 (92%) | 34/42 (81%) | 54/59 (92%) | 34/42 (81%) |
|  | GMT | 87 (56-135) | 147 (86-253) | 189 (117-305) | 229 (125-419) | 123 (80-190) | 140 (90-218) | 145 (95-222) | 75 (37-153) |
|  | % ≥1:40 | 74% (61%-84%) | 79% (64%-91%) | 83% (72%-92%) | 85% (71%-94%) | 80% (68%-89%) | 85% (71%-94%) | 83% (72%-92%) | 65% (48%-79%) |
|  | % ≥4-fold rise | 31% (20%-45%) | 47% (31%-64%) | 46% (33%-60%) | 59% (42%-74%) | 47% (34%-61%) | 50% (34%-66%) | 48% (35%-61%) | 24% (12%-39%) |

**Table S4**. Confirmed influenza positive cases following vaccination.

| **Year** | **Vaccination Group** | **Months Post-HCT at First Vaccination** | **Strain** | **Months from Dose 1 to Infection** | **Months from Dose 2 to Infection** |
| --- | --- | --- | --- | --- | --- |
| 1 | SD-QIV | 18.4 | A/H3N2 | 4.3 | 2.7 |
| 1 | SD-QIV | 15.5 | B/Victoria | 4.9 | 3.8 |
| 1 | HD-TIV | 4.1 | B/Yamagata* | 3.5 | 2.4 |
| 2 | SD-QIV | 7.3 | A/H3N2 | 2.8 | 1.0 |
| 2 | HD-TIV | 3.6 | B/Yamagata* | 1.2 | 0.2 |
| 2 | HD-TIV | 12.0 | B/Yamagata* | 2.7 | 1.3 |
| 2 | SD-QIV | 11.4 | B/Yamagata | 5.4 | 4.2 |
| 2 | HD-TIV | 5.5 | A/H3N2 | 5.3 | 4.1 |
| 2 | HD-TIV | 26.6 | B/Yamagata* | 4.7 | 3.3 |
| 3 | HD-TIV | 7.6 | A/Untypable | 2.9 | 1.8 |
| 3 | HD-TIV | 9.2 | A/H3N2 | 6.1 | 5.0 |
| 3 | SD-QIV | 6.3 | A/H1N1 | 4.4 | 3.3 |
| 3 | SD-QIV | 6.4 | A/H3N2 | 4.9 | 2.4 |

* B/Yamagata is not included in HD-TIV.
